# Nutritional Care for Older Patients Undergoing Rehabilitation

**DOI:** 10.3390/nu15132893

**Published:** 2023-06-26

**Authors:** Kae Morita, Yuka Shirai, Momoko Tohyama, Ryo Momosaki

**Affiliations:** Department of Rehabilitation Medicine, Mie University Graduate School of Medicine, Tsu 514-8507, Japan

Malnutrition is a common complication in patients undergoing rehabilitation. A previous study investigating the percentage of malnourished older patients found that the rate of malnutrition was the highest in rehabilitation facilities [1]. One of the reasons for the high prevalence of malnutrition in rehabilitation facilities is that acute illness or trauma requiring rehabilitation can lead to hypermetabolism, which can cause malnutrition [2]. Malnutrition could cause disability after conditions, such as sarcopenia, which can increase the risk of required rehabilitation [3]. Moreover, appropriate nutritional management may not be performed during acute treatment. All of these factors could increase the risk of malnutrition among patients undergoing rehabilitation.

Nutritional management has been reported to provide numerous benefits in patients undergoing rehabilitation. A combination of low-intensity exercise and nutritional supplementation has been reported to significantly increase weight gain, improve respiratory function, increase walking distance, improve quadriceps muscle strength, and reduce inflammation in patients with chronic obstructive pulmonary disease experiencing malnutrition [3]. High-intensity resistance training and whey protein supplementation have been reported to significantly improve sarcopenia in older men with reduced muscle mass [4]. A combination of whey protein supplementation and rehabilitation has been shown to be helpful in improving activities of daily living (ADL) in post-operative patients with hip fractures [5]. Intensive nutritional management with dietary supplements has been reported to improve ADLs, extend the walking distance, and increase the rate of discharge to home among patients undergoing rehabilitation after stroke [6]. Continuous consultation on nutrition and exercise has been shown to prevent weight loss in patients after stem cell transplantation [7]. Thus, rehabilitation combined with a nutritional intervention improves the prognosis in patients with various diseases. Therefore, nutritional management is critical to enhance the effects of rehabilitation.

Comprehensive nutrition screening is necessary to identify patients undergoing rehabilitation who require nutritional management. Nutrition screening tools in the rehabilitation setting include the Mini Nutritional Assessment Short-Form, Malnutrition Screening Tool, Malnutrition Universal Screening Tool, Short Nutritional Assessment Questionnaire, and Nutritional Form for the Elderly [8]. Nutrition screening tools must not only be simple and rapid, but also have diagnostic accuracy. In the rehabilitation setting, the Malnutrition Universal Screening Tool has been reported to be superior to other screening tools, with a sensitivity of 100% and specificity of 97% for malnutrition [9]. For all patients undergoing rehabilitation, such nutrition screening tools should be used to identify malnourished patients.

For patients identified by screening to be at high risk of malnutrition, the initial step should be a detailed assessment of their nutritional status. Many variations in nutritional assessment methods, such as the Mini Nutritional Assessment, Subject Global Assessment, Patient-Generated, and Objective Data Assessment, are used in patients undergoing rehabilitation across facilities [8]. Unification of nutritional assessment methods is required to identify the current status of malnourished patients in various facilities. Unification of malnutrition assessment methods for patients undergoing rehabilitation and standardization of nutrition assessment methods could improve the disparities between facilities, and further research is required.

## References

[1] Kaiser M.J., Bauer J.M., Rämsch C., Uter W., Guigoz Y., Cederholm T., Thomas D.R., Anthony P.S., Charlton K.E., Maggio M. (2010). Mini Nutritional Assessment International Group. Frequency of malnutrition in older adults: A multinational perspective using the mini nutritional assessment. J. Am. Geriatr. Soc..

[2] Jensen G.L., Mirtallo J., Compher C., Dhaliwal R., Forbes A., Grijalba R.F., Hardy G., Kondrup J., Labadarios D., Nyulasi I. (2010). International Consensus Guideline Committee. Adult starvation and disease-related malnutrition: A proposal for etiology-based diagnosis in the clinical practice setting from the International Consensus Guideline Committee. Clin. Nutr..

[3] Sugawara K., Takahashi H., Kasai C., Kiyokawa N., Watanabe T., Fujii S., Kashiwagura T., Honma M., Satake M., Shioya T. (2010). Effects of nutritional supplementation combined with low-intensity exercise in malnourished patients with COPD. Respir. Med..

[4] Kemmler W., Kohl M., Jakob F., Engelke K., von Stengel S. (2020). Effects of High Intensity Dynamic Resistance Exercise and Whey Protein Supplements on Osteosarcopenia in Older Men with Low Bone and Muscle Mass. Final Results of the Randomized Controlled FrOST Study. Nutrients.

[5] Niitsu M., Ichinose D., Hirooka T., Mitsutomi K., Morimoto Y., Sarukawa J., Nishikino S., Yamauchi K., Yamazaki K. (2016). Effects of combination of whey protein intake and rehabilitation on muscle strength and daily movements in patients with hip fracture in the early postoperative period. Clin. Nutr..

[6] Rabadi M.H., Coar P.L., Lukin M., Lesser M., Blass J.P. (2008). Intensive nutritional supplements can improve outcomes in stroke rehabilitation. Neurology.

[7] Hung Y.C., Bauer J.D., Horsely P., Coll J., Bashford J., Isenring E.A. (2014). Telephone-delivered nutrition and exercise counselling after auto-SCT: A pilot, randomised controlled trial. Bone Marrow Transp..

[8] Marshall S. (2016). Protein-energy malnutrition in the rehabilitation setting: Evidence to improve identification. Maturitas.

[9] Kruizenga H.M., Seidell J.C., de Vet H.C., Wierdsma N.J., van Bokhorst-de van der Schueren M.A. (2005). Development and validation of a hospital screening tool for malnutrition: The short nutritional assessment questionnaire (SNAQ). Clin. Nutr..

